# Chemical Profiling and Screening of the Marker Components in the Fruit of *Cassia fistula* by HPLC and UHPLC/LTQ-Orbitrap MS^n^ with Chemometrics

**DOI:** 10.3390/molecules23071501

**Published:** 2018-06-21

**Authors:** Jiawei Tan, Mengcheng Zheng, Susu Duan, Yanling Zeng, Ziwei Zhang, Qingyu Cui, Jiamei Zhang, Tingting Hong, Jie Bai, Shouying Du

**Affiliations:** School of Chinese Materia Medica, Beijing University of Chinese Medicine, Yangguang South Avenue, Fangshan District, Beijing 102488, China; tanjiawei_2017@163.com (J.T.); zheng0221109@163.com (M.Z.); 18810980870@163.com (S.D.); zengyl@bucm.edu.cn (Y.Z.); 18810689515@163.com (Z.Z.); qingyu.cui@outlook.com (Q.C.); zhangjm370@163.com (J.Z.); hongtingting1995@163.com (T.H.)

**Keywords:** the fruit of *Cassia fistula*, UHPLC/LTQ-Orbitrap MS^n^, source differences, similarity analysis (SA), chemometrics

## Abstract

*Cassia fistula* L. which is known as “Golden Shower”, is used as an ornamental plant due to its flowers, and fruit parts of this plant have a high medicinal value. There are few reports providing a comprehensive overview of the chemical composition of its fruit or explaining the differences between samples from different sources because of the complexity of its chemical components. The purpose of the present study was to establish a fingerprint evaluation system based on Similarity Analysis (SA), Hierarchical Cluster Analysis (HCA) and Principal Component Analysis (PCA) for the composition identification and quality control of this herb. Twelve samples from Xinjiang and Sichuan provinces in China and India were analyzed by HPLC, and there were fifteen common peaks in the twelve batches. Molecular weight and formula information can be derived from thirty-one peaks by UHPLC/LTQ-Orbitrap MS^n^, molecular structure information of twenty components was obtained, of which ten compounds were identified by comparison with standard materials. Samples of twelve batches were divided according to their similarity into four groups, which were basically consistent with three different *C.* *fistula* fruit-producing areas. Five compounds were finally considered to be chemical markers to determine the quality of this herb. A fingerprints method combined with chemometrics was established to differentiate the origin of the fruit of *C. fistula* which has the advantages of effectivity and convenience, laying the foundation for the quality evaluation of this herb from different sources.

## 1. Introduction

*Cassia fistula* Linn. (Caesalpinaceae) which is native to India, the Amazon and Sri Lanka, is a semi-wild Indian Labernum also commonly known as “golden shower”, which is extensively cultivated throughout India as an ornamental and deciduous plant for its beautiful bunches of yellow flowers. The plant has been widely diffused in tropical countries around the world, from the West Indies to South Asia and from South Africa to Egypt, Mauritius, India, South Africa, Mexico, China, East Africa, Brazil, and so on [1,2,3]. “Golden shower” not has only a high ornamental value, but also possesses useful properties in the treatment of diseases. *C. fistula* has been used as a kind of traditional effective drug by local people for a long time. It has been explained in the Indian literature that the plant has many advantageous applications against some diseases such as skin infections, liver troubles, tuberculous glands, and even malaria [4]. One of the most representative is its fruit parts which was reported to have a high medicinal utility. Fruit parts of *C. fistula* have been reported scientifically to possess hypolipidemic, antidiabetic, antioxidant, antimicrobial, antitumor, sedative, purgative, hepatoprotective and abortifacient activity [5,6,7,8,9,10,11].

It has generally acknowledged that the efficacy of plant drugs is related to their chemical composition and active compounds. At present, more studies have focused on the pharmacological and biological activities of plants’ individual organs. Few studies have provided a comprehensive description of the chemical profile and explained the differences between samples from different origins because the chemical composition of the fruit of *C. fistula* is so complicated [12,13,14,15,16,17]. Because of the lack of identification of subsequent peaks, one cannot point out the specific components which cause these differences. Therefore, it is very necessary and important to establish a valid strategy to comprehensively identify the chemical components of the fruit of *C. fistula*, and to seek several characteristic markers which can be applied to discriminating the medicinal herbs from different places of production and control their quality.

In this case, HPLC fingerprints of critical components provide a new approach for quality control of drugs. There are numerous studies about fingerprint analysis combined with chemometrics for the screening of bioactive components and the quality control of medicinal drugs [18,19,20]. In the present research, thirty-six peaks were detected by HPLC, of which fifteen were common peaks in the similarity analysis (SA). In order to identify clearly what these peaks are, we adopted a UHPLC/LTQ-Orbitrap MS^n^ method. It has the advantages of high resolution and sensitivity, and HPLC coupled with hybrid LTQ-Orbitrap MS system has been applied to many studies in this field [21,22,23], because it is an ideal identification method for natural compounds by obtaining accurate molecular mass and multistage MS^n^ fragment ions of the tested samples. Screening a representative sample (S7) that can reflect the peak characteristics of these twelve batches of medicinal materials as far as possible by UHPLC/LTQ-Orbitrap MS^n^. UHPLC/LTQ-Orbitrap MS^n^ was performed using the negative ion mode of the HESI source for detection, molecular weight and formula information was gained from a total of thirty-one peaks, the molecular structure information of twenty components was obtained through matching their accurate molecular weights with known compounds in the online databases and by comparison of their molecular ions and fragment ions, whereby ten compounds were identified. Moreover, three components were discovered for the first time in the fruit of *C. fistula*, two of these compounds are related to sennoside A/B and one is a new compound. Another compound, 1-[1,5-dihydroxy-3-methyl-8-[3,4,5-trihydroxy-6-[(3,4,5-trihydroxyoxan-2-yl)oxymethyl]oxan-2-yl]oxynaphthalen-2-yl]ethanone (PubChem CID: 22297273), was also reported for the first time in this herb. To the best of our knowledge, it is the first time that so many compounds were inferred in the fruit parts of *C. fistula.* Differences in medicinal materials result from the number and content of ingredients, so for the purpose of seeking the reasons that may lead to differences between several batches, we need to study these components with chemometrics. According to the HCA result the samples were divided into four groups that approximately coincided with three different places of origin of the fruit of *C. fistula*. We also found for the first time that these compounds, including C_12_H_22_O_11_ (disaccharide), C_17_H_30_O_12_, C_27_H_30_O_15_, C_26_H_28_O_14_ and C_15_H_22_O_2_, can be suitable markers to discriminate between these medicinal materials of different quality according to PCA. In conclusion, the results proved that fingerprints combined with chemometrics represent an effective, simple and rapid means for the quality control of *C. fistula* fruit.

## 2. Results and Discussion

### 2.1. Validation of the Method

Fingerprinting is a comprehensive and quantifiable means of identification. It is based on the systematic study of pharmaceutical chemical components and is mainly used to evaluate the authenticity, superiority and stability of drugs. The relative retention time, relative peak area and similarities were used to evaluate the quality of the fingerprints. Rhein (peak t) which is a relatively large single peak in the chromatogram, was assigned as the reference peak to calculate relative retention times and relative peak areas.

Precision testing was performed by repeated injection of the same sample solution six continuous times. The RSD of relative retention time of the common peaks was below 0.33%, the RSD of relative peak area was below 4.84%, and the similarities of six chromatograms were all 1.000.

The stability was evaluated with one sample over 24 h. The RSDs of relative retention time and relative peak area of the common peaks were all below 0.99% and 3.29%, and the similarities of different chromatograms were all above 0.999.

The repeatability was determined by the detection of six prepared samples. The RSDs of relative retention time and relative peak area of the common peaks were respectively below 0.80% and 4.54%; the similarities of six chromatograms were all above 0.999.

These results showed that all samples remained stable during the testing period, and demonstrated the conditions were reliable and satisfactory for the fingerprint analysis.

### 2.2. Similarity Analysis (SA)

The chromatographic profile must be representative of all samples and characteristics of integrity and fuzziness. The identification and analysis of the samples can be conducted well even though the amounts of several chemical components are different from the others by analyzing the mutual pattern of chromatograms [24].

**Figure 1:**
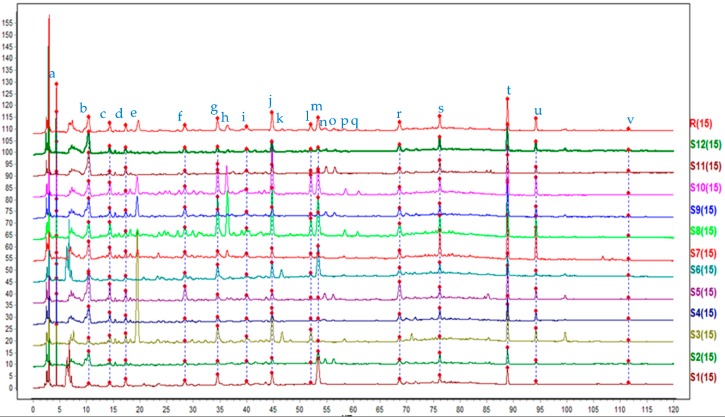
Covered HPLC chromatograms of samples from No. S1 to S12. The common pattern (marked R) was obtained by SES.

Twelve batches of samples from different origins (Table 1) were determined and the chromatograms were analyzed by SES to generate a common pattern R (Figure 1). Peak a, b, c, d, f, g, i, j, l, m, r, s, t, u and v were common peaks, and their peak areas are listed in the Supplementary Materials. The SES software was used to calculate the similarities of different chromatograms compared to the common pattern. All these results are shown in Table 2.

From the results we can conclude that the similarities of the different chromatograms compared to the common pattern were all above 0.800, except for sample S1 (0.796), which indicated that chemical constituents of different samples were not highly influenced by their origins. The common pattern is a very effective identification for the samples of the fruit of *C. fistula.*

Other than these fifteen common peaks, we can also draw the following conclusions from the chromatograms: peak e appeared in S3, S7, S8, S9 and S10, among which the peaks of S3, S9 and S10 had a relatively large peak area; peak h appeared in S7, S8, S10 and S12, and the peak areas of S8 and S10 were relatively larger; peak k merely appeared in S3 and S6; both peak n and o appeared in S2, S5, S9 and S11; both peak p and q only appeared in S8 and S10. The uncommon peaks of these respective batches were also responsible for differences in these medicinal materials from different sources. From these uncommon peaks, we can find out clearly that the samples of S3, S8 and S10 were different from other samples, and this result can be linked to that of Hierarchical Cluster Analysis (HCA) described below to elaborate the differences. To find out what the common peaks screened are by SA, we need to identify them carefully.

### 2.3. Chemical Profiling of the Fruit of C. fistula by UHPLC/LTQ-Orbitrap MS^n^

Liquid chromatography coupled with mass spectrometry has been proved as a powerful tool for structural characterization of unknown compounds [25]. There are relatively few reports on the composition of the fruit parts of *C. fistula*, so it is especially meaningful to identify the constituents by high-resolution mass spectrometry. We can obtain the total ion current (TIC) chromatogram of methanol extract of the fruit of *C. fistula* in negative ion mode by UHPLC/LTQ-Orbitrap MS^n^. Since most of ingredients of the medicinal drug are flavonoids and anthraquinones, mainly acidic components, we mainly analyzed the negative ion mode. Firstly, the chemical compounds of the fruit of *C. fistula* were systematically investigated and summarized from previous reference literatures. Secondly, the databases (e.g., ChemSpider website http://www.chemspider.com and PubChem https://pubchem.ncbi.nlm.nih.gov/) were employed to search for compound structures based on the predicted chemical formulae. Finally, the structure of the compound is tentatively or unequivocal deduced by its chromatographic behavior, MS fragment data, comparisons with reference standards, and database-matching (e.g., Metlin https://metlin.scripps.edu/landing_page.php?pgcontent=mainPage). These compounds identified are classified into three groups including flavonoids, anthraquinones, disaccharide and others, the first two being the main ones.

**Figure 2:**
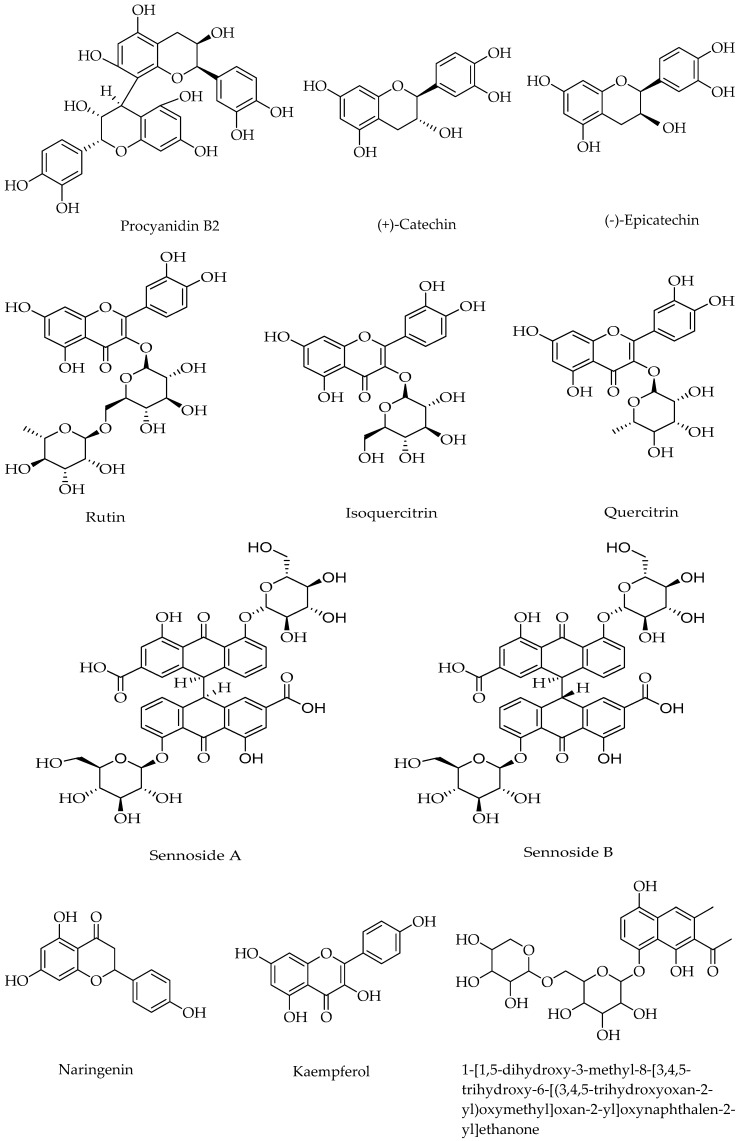

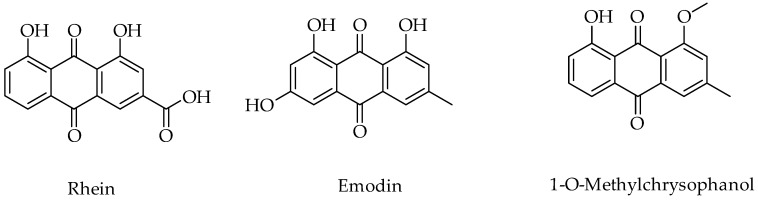
Chemical structure of fourteen compounds that were tentatively identified.

**Figure 3:**
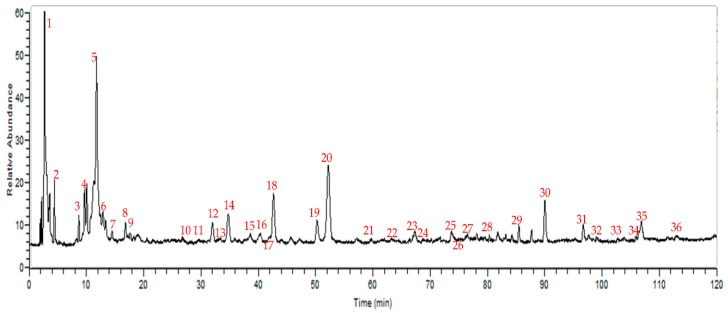
Total ion chromatogram of the fruit of *C. fistula* in negative-ionization mode.

We selected a representative sample (S7) of twelve batches of these medicinal drugs that can reflect the peak characteristics as far as possible by UHPLC/LTQ-Orbitrap MS^n^. Altogether, there were a total of thirty-six peaks, the [M − H]^−^ ions can be found for thirty-one compounds, the molecular structure information was acquired for twenty components, and ten components were accurately identified by comparison with standard substances. Some of these compounds are presented in Figure 2. In addition, a new compound had been found (sennoside triglucoside), another other compound (sennoside monoglucoside) had been deduced in the fruit of *C. fistula* for the first time among the sennoside derivatives, and another compound, 1-[1,5-dihydroxy-3-methyl-8-[3,4,5-trihydroxy-6-[(3,4,5-trihydroxyoxan-2-yl)oxymethyl]oxan-2-yl]oxynaphthalen-2-yl]ethanone was also reported for the first time in this herb. Figure 3 shows the total ion current (TIC) chromatogram of methanol extract of this medicinal drug in the negative mode by UHPLC/LTQ-Orbitrap MS^n^. Detailed information about this drug is given in Table 3, considering the elution order.

**Figure 4:**
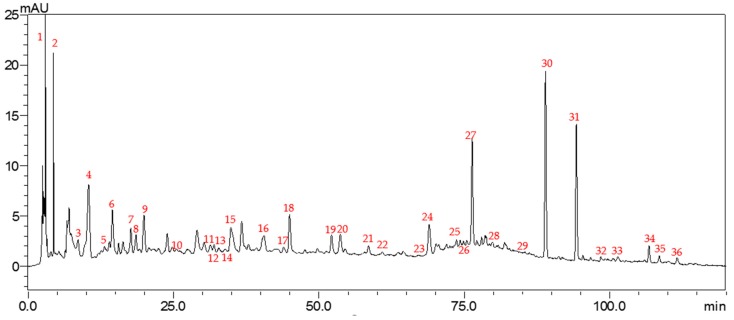
HPLC chromatogram of the fruit of *C. fistula*.

The HPLC chromatogram profiles (Figure 4) also basically correspond to that of the UHPLC/LTQ-Orbitrap MS^n^ chromatogram of the fruit of *C. fistula* in negative-ionization mode, in both of which thirty-six chromatographic peaks were detected.

#### 2.3.1. Identification of Flavonoids

**Figure 5:**
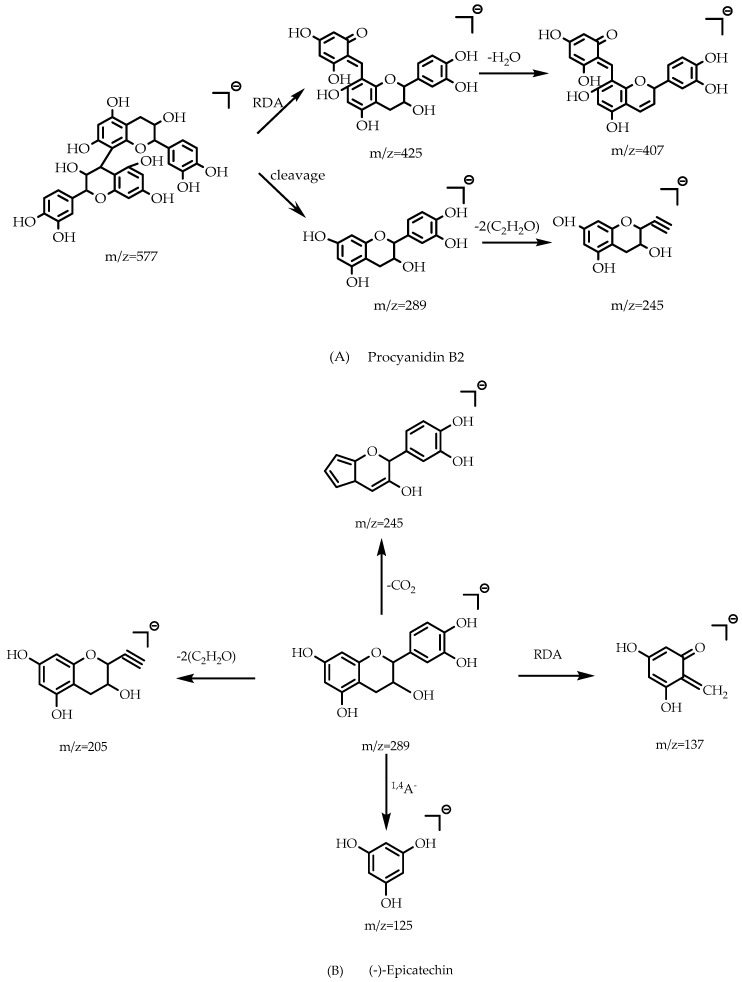

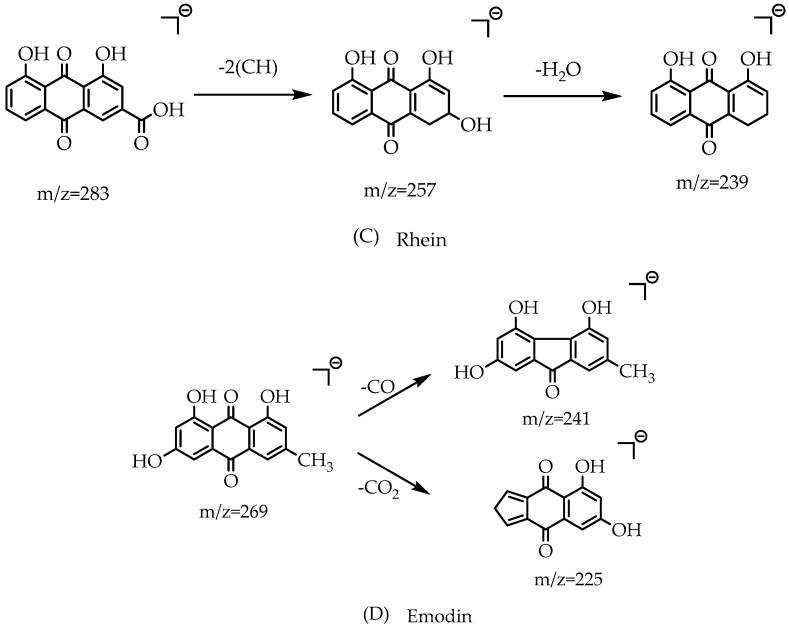
RDA cleavage and binding sites of four components.

Procyanidins are a type of polymers consisting of a flavane-3-alcohol monomer as the basic unit. Among them, type B procyanidins are the most widely distributed. Compound **5** was provisionally assigned as procyanidin B2 due to its parent ion [M − H]^−^ at *m*/*z* 577 and predominant daughter ions at *m*/*z* 425 and 407 because of a RDA cleavage, and at *m*/*z* 289 and 245, indicating the loss of procyanidin B2 in agreement with its MS/MS data of the known compound from the literature. Figure 5A shows the RDA cleavage and binding sites of procyanidin B2 [26].

Compounds **6** and **8** were assigned as (+)-catechin and (−)-epicatechin, respectively. The two components are isomers of each other. Compound **6** was tentatively identified as (+)-catechin because of its parent ion [M − H]^−^ at *m*/*z* 289 and corresponding fragment ions at *m*/*z* 245 and 205, and at *m*/*z* 137 (due to the RDA cracking method) and 125. It was characterized as (+)-catechin after comparing its MS/MS data with this compound through the search of the literature and databases, and compound **8** was similarly deduced. Figure 5B shows the RDA cleavage and binding sites of (−)-epicatechin [26].

Compounds **11**, **13** and **17** are quercetin derivatives, which that are the major flavonoids in the fruit of *C. fistula* under investigation, according to their common fragment ion at *m*/*z* 301 which is characteristic of quercetin. Because of the removal of glucose and rhamnose, respectively, they were therefore hypothetically assigned to be isoquercitrin (**13**) and quercitrin (**17**). Compound **11** was putatively identified as rutin because of the similarity with spectral library data.

Peak **20** gave a [M − H]^−^ ion at *m*/*z* 563 and produced fragment ions at *m*/*z* 299 and *m*/*z* 255 (yielded after losing 44 Da of CO_2_), suggesting a formula of C_26_H_28_O_14_, it was hard to understand how the *m*/*z* 299 ion can be produced, and it was assumed that it came from the loss of two molecules of xylose.

Compounds **24** and **28** are kaempferol derivatives which are the other relatively major flavonoids in the fruit of *C. fistula* under research, in the light of the quasi-molecular ion of compound **28** at *m*/*z* 285 that is characteristic of kaempferol, and its fragment ions at *m*/*z* 257 and 211 after the comparison of its MS/MS data. The fragment ions of compound **24** are approximately similar to the fragment ions of kaempferol, so compound **24** can be considered as its isomer.

Compound **27** had a quasi-molecular ion at *m*/*z* 271. The C ring of this compound underwent RDA cleavage to generate fragment ions of *m*/*z* 151 and 119, and the B ring was cracked to generate fragment ions with *m*/*z* l77 and 93, so this component was presumed to be naringenin by comparison of its molecular ions and fragment ions with literature values.

#### 2.3.2. Identification of Anthraquinones

Compounds **12**, **15**, **18** and **26** are sennoside derivatives, which are the typical diterpenoids in the fruit of *C. fistula* under study, based on their common fragment ions at *m*/*z* 537 that are characteristic of sennoside aglycon. Compounds **15** and **18** were temporarily confirmed as sennoside B and sennoside A separately by comparison to a known standard with the same fragmentation pattern. The *m*/*z* 861, 699 and 537 fragment ions were presumed to have lost glucose (162 Da). It was estimated that compound **12** was a new component which consisted of sennoside aglycon linked with three molecules of glucose, since the compound could not be found in multiple databases. Moreover, compound **26** gave an [M − H]^−^ ion at *m*/*z* 699 and produced a fragment ion at *m*/*z* 537 after losing 162 Da of one molecule of glucose, suggesting a formula of C_36_H_28_O_15_ and was inferred to consist of sennoside aglycon linked with one molecule of glucose (9-[2-carboxy-4-hydroxy-10-oxo-5-[(2S,3R,4S,5S,6R)-3,4,5-trihydroxy-6-(hydroxymethyl)oxan-2-yl]oxy-9H-anthracen-9-yl]-4,5-dihydroxy-10-oxo-9H-anthracene-2-carboxylic acid, PubChem CID: 10101233). The exact structure of these two compounds is yet to be further confirmed. The secondary fragment ion chromatograms of the two components are presented in the Supplementary Materials.

Free anthraquinones are typical of anthraquinones, and the following five free anthraquinones are the most common: rhein, emodin, chrysophanic acid, aloe emodin, physcion. Rhein and emodin were inferred to exist after comparing these compounds with the literature. Compound **30** was tentatively identified as rhein because of its parent ion [M − H]^−^ at *m*/*z* 283 and daughter ion at *m*/*z* 257 and the other one at *m*/*z* 239. According to the prediction of the mass spectrometry, the fragment ion at *m*/*z* 257 should lose a group with a mass of about 26 from the quasi-molecular ion, but there was no a mass of approximately 26 in this system. The analysis showed it should be a system where the carboxyl of the quasi-molecular ion system lost a CO-neutral small molecule with a mass number of 28, but it was also affected by the H radical in the ion trap. One of the H radicals combined with its oxygen atom to form a hydroxyl at the cleavage site of losing a CO molecule, and the other H radical combined with its adjacent carbon at the cleavage site to form a *m*/*z* 257 fragment ion. The formation of *m*/*z* 239 fragment ion was due to that the formed hydroxyl underwent a dehydration reaction with the H in its *ortho* position. Compound **33** was deduced as emodin due to its [M − H]^−^ ion at *m*/*z* 269 and the diagnostic ion at *m*/*z* 241 which underwent a loss of CO radical, and *m*/*z* 225 from losing a molecule of CO_2_. Compound **31** was tentatively assigned as 1-O-methylchrysophanol because of the deprotonated ion [M − H]^−^ at *m*/*z* 269 and the fragment ions at *m*/*z* 254 and 226 due to the similarity with the spectral library data. Figure 5C,D show the RDA cleavage and binding sites of rhein and emodin respectively [27]. Compound **25** can be considered as 1,3,8-trihydroxy-6-methoxyanthraquinone in view of its [M − H]^−^ at *m*/*z* 285 and daughter ions at *m*/*z* 270 and 241 through analyzing its molecular structure.

#### 2.3.3. Identification of Disaccharides and Other Compounds

Compounds **1** and **2** were inferred to be disaccharides in the fruit of *C. fistula* based on their common fragment ion at *m*/*z* 179 which is characteristic of aldohexoses. The fruit of *C. fistula* is a bit sticky, and it was suspected that there are more saccharides in it. The method is not suitable for the analysis of polysaccharides inside, and they can be detected through more advanced methods.

Compound **14** was tentatively assigned as 1-[1,5-dihydroxy-3-methyl-8-[3,4,5-trihydroxy-6-[(3,4,5-trihydroxyoxan-2-yl)oxymethyl]oxan-2-yl]oxynaphthalen-2-yl]ethanone. This compound, whose parent ion [M − H]^−^ was at *m*/*z* 525 and predominant daughter ion was at *m*/*z* 231, first lost a molecule of xylose and glucose combined with its secondary mass spectrometry fragment ion information. When the MS^3^ scan was performed, it also took off an acetyl molecule according to its tertiary mass spectrometry fragment ions at *m*/*z* 189 and 187. Its chemical structure is shown in Figure 2, and the secondary and tertiary fragment ion chromatograms of this compound are listed in the Supplementary Materials.

Peaks **3**, **4**, **7**, **16**, **20**, **23**, **29**, **32**, **34**, **35** and **36** are currently only defined by their molecular formulas and molecular weights, and their structural information needed to be further confirmed by other approaches such as NMR.

As a result, the molecular formulas of thirteen peaks were determined for fifteen common peaks in Figure 1, they were as follows: C_12_H_22_O_11_ (Peak a), C_17_H_30_O_12_ (Peak b), C_15_H_14_O_6_ (Peak c), C_27_H_30_O_15_ (Peak d), C_42_H_38_O_20_ (Peak g), C_30_H_26_O_9_ (Peak i), C_42_H_38_O_20_ (Peak j), C_26_H_28_O_14_ (Peak m), C_15_H_10_O_6_ (Peak r), C_15_H_12_O_5_ (Peak s), C_15_H_8_O_6_ (Peak t), C_15_H_10_O_5_ (Peak u), C_15_H_22_O_2_ (Peak v). Among these thirteen formulas, the structure of eight peaks has been putatively identified respectively as a disaccharide (Peak a), (+)-catechin (Peak c), sennoside B (Peak g), sennoside A (Peak j), the isomer of kaempferol (Peak r), naringenin (Peak s), rhein (Peak t), 1-O-methylchrysophanol (Peak u).

### 2.4. Identification of Ten Chemical Components by Comparison with Standard Materials

**Figure 6:**
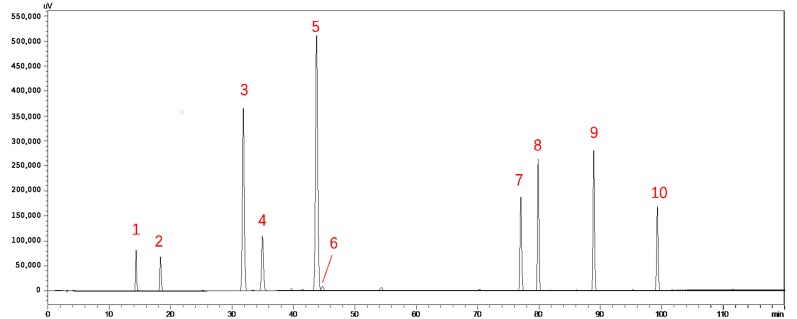
HPLC chromatogram of ten standard materials (1. (+)-catechin; 2. (−)-epicatechin; 3. rutin; 4. sennoside B; 5. quercitrin; 6. sennoside A; 7. naringenin; 8. kaempferol; 9. rhein; 10. emodin).

**Figure 7:**
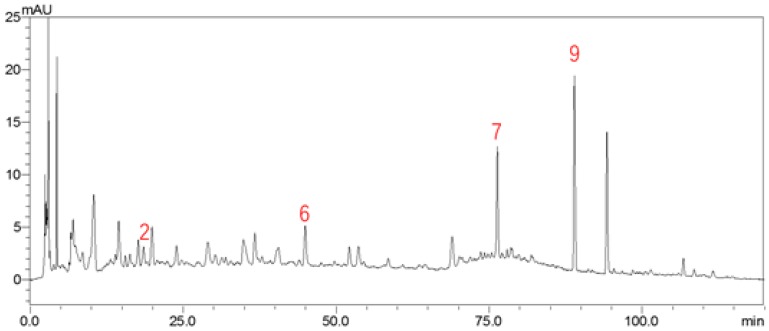
HPLC chromatogram of the fruit of *C. fistula* involving four quantitative components (2. (−)-epicatechin; 6. sennoside A; 7. naringenin; 9. rhein).

To accurately confirm more components, the following compounds were compared with standard materials: (+)-catechin, (−)-epicatechin, rutin, sennoside B, quercitrin, sennoside A, naringenin, kaempferol, rhein and emodin (Figure 6). Since these compounds were reported several times to have biological activities, they were quantified by means of chromatography. Comparing the chromatogram of the medicinal material with that of these standard materials, separation and tailing of four compounds, (−)-epicatechin (**2**), sennoside A (**6**), naringenin (**7**), and rhein (**9**), met the requirements, so they can be used as quantitative indicators. This method provided the basis for establishing its quality standards, and the result is shown in Figure 7.

### 2.5. Hierarchical Cluster Analysis (HCA)

In order to evaluate resemblances and dissimilarities of the fruits of *C. fistula* based on the fingerprint data, HCA was performed which can divide tested samples into different groups [20]. HCA is a useful and simple statistical method for seeking relatively homogeneous clusters based on measured characteristics, and samples with high similarity can be clustered into the homogenous groups. Recently, this method is widely used in the origin discrimination, identification, quality assessment of drugs, and is diffusely applied to the fingerprint analysis [28]. The method can classify different medicinal herbs by measuring the peak areas from their corresponding HPLC fingerprints. The common characteristic peaks, which were calculated by the SES, were selected for the HCA. HCA of the samples was performed based on the relative peak areas of all fifteen common peaks.

**Figure 8:**
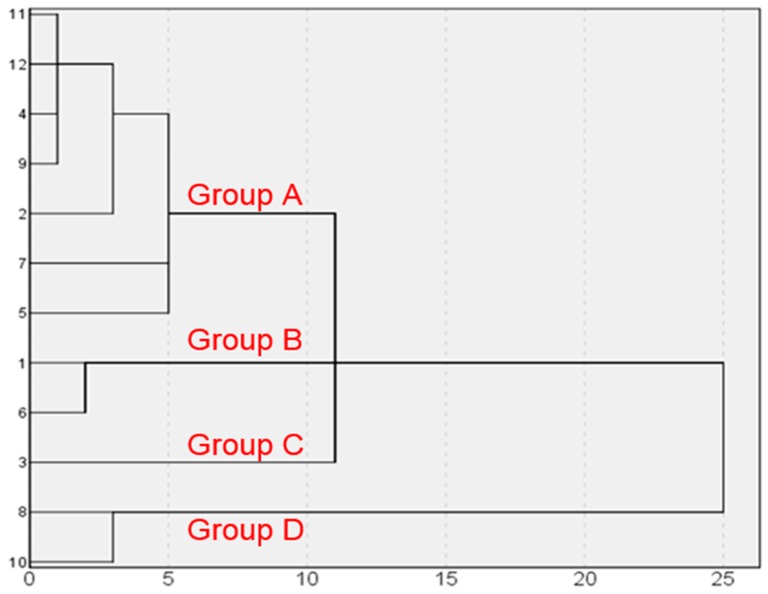
Results of Hierarchical Cluster Analysis of twelve samples.

The HCA results are shown in Figure 8, where the quality characteristics were revealed clearly, and the results revealed that the samples can be divided into four quality clusters. Interestingly, these four groups were basically similar to the three different producing areas (shown in Table 1) of these herbs, including Xinjiang Province (Group A and C) in China, India (Group B) and Sichuan Province (Group D) in China. Group A was made up of S2, S4, S5, S7, S9, S11 and S12; Group B consisted of S1 and S6; Group C was made up of S3; Group D was composed of S8 and S10. The case which divided into four groups indicated that there were differences in chemical species and content among India, Xinjiang Province and Sichuan Province samples. Overall, it would be more intuitive and effective to distinguish the fruit of *C. fistula* from different regions according to the results of the HCA.

### 2.6. Principal Components Analysis (PCA)

The HPLC response signal from fifteen characteristic components of twelve batches of the fruit of *C. fistula* was imported into SPSS 25.0 software (SPSS, Chicago, IL, USA) with their peak areas as the tested object. PCA provided an easy visualization of the complete data set in a reduced dimension plot, showing the degree of aggregation and dispersion during several components of different samples, and the method was used to explain the differences [29].

**Figure 9:**
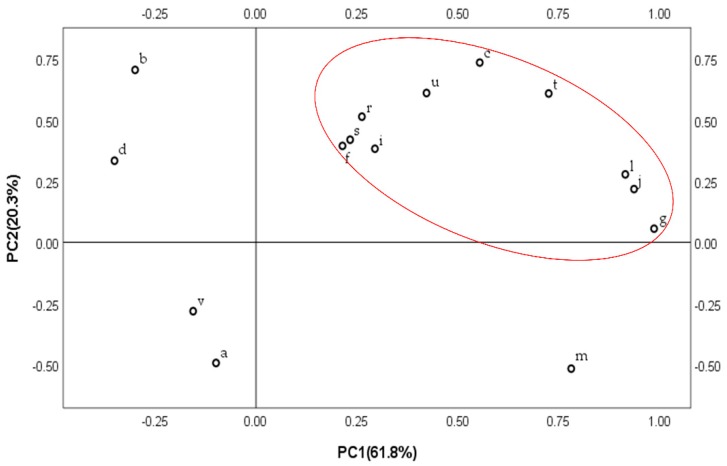
PCA loading plots of the sample from different sources.

The first two principal components in the correlation plot (SPSS 25.0) were extracted by PCA, and the cumulative contribution of variances of these components was up to 82.1%. PC1 represented 61.8% of the total variance, and it had the highest contribution rate and contained the most information; PC2 represented 20.3% of the total variance. The factor loading matrix of tested samples is shown in Table 4, and the loading plots of PCA of fifteen components of the fruit of *C. fistula* are shown in Figure 9 [20,24,30,31,32].

The factor loads can reflect the contribution of every index to the principal component. The sign of the factor load indicated the effect of each index on the change of the principal component value. According to the results it can be seen that peaks g, j and l had a significant positive phase load in the first principal component, indicating that the value of the first principal component increases as the three peaks increase, and peaks a, b, d and v had an inverse load in the first principal component; Peaks b and c with positive phase load were the relatively main determinants, and peaks a, m and v had a relative inverse load in the second principal component. The loading plots of the PCA model can explain the contribution of variables to each principal component. Most of the compounds were comparatively clustered together, and five compounds were outliers that were responsible for the significant differences in these medicinal materials from different places. Combination of the factor loading matrix and loading plots indicated that the main components responsible for the separation were C_12_H_22_O_11_ (disaccharide, peak a), C_17_H_30_O_12_ (peak b), C_27_H_30_O_15_ (peak d), C_26_H_28_O_14_ (peak m) and C_15_H_22_O_2_ (peak v) which contributed most to the grouping result. These five compounds were much more statistically significant in chemotaxonomy than the other identified components, and the higher concentration of them may be due to the good quality of the fruit of *C. fistula.*

## 3. Materials and Methods

### 3.1. Reagents and Materials

A total of twelve batches of the fruit of *C. fistula* collected in present study were purchased from various corporations and hospitals from two provinces in China. The batch numbers are listed in Table 1. Reference compounds of (+)-catechin (877-200001), (−)-epicatechin (878-200102), rutin (100080-201408), quercitrin (111538-200302), rhein (110757-201607) and emodin (110756-201512) were obtained from the National Institutes for Food and Drug Control (Beijing, China), and sennoside A (81-27-6), sennoside B (128-57-4), kaempferol (520-18-3) and naringenin (480-41-1) were obtained from Yuanye Bio-Technology Corporation (Shanghai, China). Purified water from Wahaha Corporation (Hangzhou, China) was used in experiments. HPLC/MS-grade acetonitrile and formic acid were purchased from Sigma-Aldrich Corporation (Shanghai, China); HPLC-grade acetonitrile, phosphoric acid and methanol were purchased from Fisher Corporation (Shanghai, China). All other reagents and chemicals used were of analytical grade.

### 3.2. Preparation of Standard Solutions and Sample Solutions

Individual stock solutions of the references used for qualitative analysis were prepared by dissolving the each reference in methanol at an appropriate concentration and then stored at 4 °C until use. The dry plant material was firstly ground into powder and sieved (65 mesh). A total of 1 g plant material powder was accurately weighed, and then extracted by ultrasonication (KQ5200DA, 200 W, 40 kHz, Kunshan, China) for 90 min with 100 mL pure methanol at room temperature for each sample. The samples were shaken well, filtered, and the filtrate was passed through a 0.45 μm microporous membrane to prepare the tested samples.

### 3.3. Instrumentation and Chromatographic Conditions

#### 3.3.1. Chemical Profiling by UHPLC/LTQ-Orbitrap MS^n^

Chromatographic analysis was performed on a Dionex Utimate 3000 Series HPLC system (Thermo Scientific, Shanghai, China) equipped with a quaternary solvent delivery system and a column temperature controller. All the samples were analyzed at a column temperature of 30 °C on a Diamosil-C_18_ column (4.6 mm × 250 mm, 5 μm, Dema, Beijing, China). The mobile phase consisted of water with 0.1% formic acid solution (eluent B), and acetonitrile (eluent A) using a gradient elution mode of 10% A at 0–3 min, 10–15% A at 3–5 min, 15–26% A at 5–60 min, 26–66% A at 60–110 min, 66% A at 110–120 min. The flow rate was 1.0 mL/min, and the injection volume was 10 μL.

A mass spectrometer (Thermo Scientific, LTQ-Orbitrap XL, Shanghai, China) was applied to MS detection. The operation conditions were as follows: capillary temperature, 350 °C; apci vaporizer temperature, 300 °C; sheath gas flow, 30 L/min; aux gas flow, 10 L/min; source voltage, 3 kV; capillary voltage, −35 V; Tube Lens, −110 V. Mass spectra were recorded across the range of *m*/*z* 50–2000. Thermo Workstation Acquisition Software Xcalibur Version 2.2 and UHPLC analysis Software Chromeleon Version 7.0 were utilized for system control, data acquisition and data processing.

#### 3.3.2. Similarity Analysis and Qualitative Analysis of Ten Standard Materials by HPLC

Similarity analysis and qualitative analysis were carried out on a LC-20AD Series HPLC system (Shimadzu, Hong Kong, China) equipped with a quaternary solvent delivery system and a column temperature controller. Chromatographic separation was conducted on a Diamosil-C_18_ column (4.6 mm × 250 mm, 5μm, Dema, Beijing, China). The mobile phase was composed of solvent B (0.1% phosphoric acid solution) and solvent A (acetonitrile) with a gradient elution program: 0–3 min, 10% A; 3–5 min, 10–15% A; 5–60 min, 15–26% A; 60–110 min, 26–66% A; 110–120 min, 66% A. The constant flow rate was 1.0 mL/min and the column was maintained at 30 °C. The injection volume was 20 μL and the detection wavelength was set at 254 nm. The PDA detector was set to scan from 190 nm to 400 nm, with 254 nm optimized as the detection wavelength for analysis.

### 3.4. Data Analysis and Statistics

The recognized components were assigned by comparison with the exact molecular weight and fragment ion peaks with the established database. Some of the proposed components were confirmed by comparison with standard substances. The HPLC data were analyzed through the Similarity Evaluation System (SES) for the Chromatographic Fingerprints of TCMs (2012), software. Hierarchical Cluster Analysis (HCA) and Principal Components Analysis (PCA) were performed by SPSS (SPSS Statistical Software Package, version 25.0, Chicago, IL, USA).

## 4. Conclusions

The therapeutic effects of medicinal drugs are based on their complicated chemical constituents, HPLC and UHPLC/LTQ-Orbitrap MS^n^ fingerprint analysis combined with chemometrics were applied to study the complex system of the fruit of *C. fistula*. In summary, a common pattern was established by determining and comparing the fingerprints of twelve samples of the fruit of *C. fistula* from different sources, and fifteen common peaks were selected. Therefore, it is very necessary to identify these peaks. A total of thirty-one components were recognized by UHPLC/LTQ-Orbitrap MS^n^, of which twenty compounds were tentatively inferred by comparing mass spectrometry data with that of reference compounds and literature data. Additionally, ten components were identified by comparison with standard materials. Furthermore, HCA and PCA can provide a good reference for quality analysis and origin research of this herb. Twelve batches of medicinal materials from different origins were categorized, and five ingredients, C_12_H_22_O_11_ (disaccharide), C_17_H_30_O_12_, C_27_H_30_O_15_, C_26_H_28_O_14_, C_15_H_22_O_2_ included, were proved to be the intuitive cause of their differences. Moreover, a new compound had been inferred (sennoside triglucoside) with regard to sennoside derivatives, and another compound had been deduced in the fruit of *C. fistula* (sennoside monoglucoside) for the first time. Another compound (1-[1,5-dihydroxy-3-methyl-8-[3,4,5-trihydroxy-6-[(3,4,5-trihydroxyoxan-2-yl)oxymethyl]oxan-2-yl]oxynaphthalen-2-yl]ethanone) was also first reported in this herb.

On the one hand, the results of multivariate statistical analysis here were not systematic because of the limitation of the number of samples collected. On the other hand, since the final elution gradient of acetonitrile was only up to 66% in this experiment, the larger polarity compounds were isolated but low polarity compounds were not isolated. Subsequent experiments will research the chemical identification of low polar components of the fruit of *C. fistula* and further study their systematic analysis.

## Figures and Tables

**Table 1:** Twelve batches of the fruit of *C. fistula*.

Samples	Origins	Specific Sources
S1	India	Gansu Foci in Medicinal Materials Limited Corporation
S2	Xinjiang, China	Xinjiang Pamir Uygur Pharmaceutical Limited Corporation
S3	Xinjiang, China	Fu’antang Native Product’s Distribution Department of Hualing Market in Shuimogou District
S4	Xinjiang, China	Xinjiang Uygur Medical Hospital
S5	Xinjiang, China	Xinjiang Pamir Uygur Pharmaceutical Limited Corporation
S6	India	Gansu Foci in Medicinal Materials Limited Corporation
S7	Xinjiang, China	Xinjiang Yinduolan Uygur Medical Limited Corporation
S8	Sichuan, China	Xinjiang Uygur Medical Hospital
S9	Xinjiang, China	Xinjiang Pamir Uygur Pharmaceutical Limited Corporation
S10	Sichuan, China	Xinjiang Uygur Medical Hospital
S11	Xinjiang, China	Xinjiang Pamir Uygur Pharmaceutical Limited Corporation
S12	Xinjiang, China	Xinjiang Pamir Uygur Pharmaceutical Limited Corporation

**Table 2:** The results of similarities of the chromatograms from different origins.

No.	S1	S2	S3	S4	S5	S6	S7	S8	S9	S10	S11	S12	R
S1	1.000	0.827	0.602	0.598	0.514	0.963	0.582	0.847	0.610	0.766	0.482	0.550	0.796
S2	0.827	1.000	0.590	0.894	0.853	0.861	0.821	0.801	0.881	0.767	0.864	0.898	0.927
S3	0.602	0.590	1.000	0.727	0.598	0.602	0.723	0.851	0.725	0.896	0.563	0.641	0.830
S4	0.598	0.894	0.727	1.000	0.953	0.632	0.932	0.737	0.978	0.754	0.933	0.970	0.929
S5	0.514	0.853	0.598	0.953	1.000	0.573	0.845	0.642	0.962	0.660	0.934	0.960	0.864
S6	0.963	0.861	0.602	0.632	0.573	1.000	0.624	0.908	0.657	0.836	0.551	0.619	0.846
S7	0.582	0.821	0.723	0.932	0.845	0.624	1.000	0.748	0.934	0.754	0.806	0.874	0.894
S8	0.847	0.801	0.851	0.737	0.642	0.908	0.748	1.000	0.752	0.978	0.606	0.691	0.929
S9	0.610	0.881	0.725	0.978	0.962	0.657	0.934	0.752	1.000	0.763	0.902	0.954	0.933
S10	0.766	0.767	0.896	0.754	0.660	0.836	0.754	0.978	0.763	1.000	0.637	0.717	0.926
S11	0.482	0.864	0.563	0.933	0.934	0.551	0.806	0.606	0.902	0.637	1.000	0.986	0.838
S12	0.550	0.898	0.641	0.970	0.960	0.619	0.874	0.691	0.954	0.717	0.986	1.000	0.900
R	0.796	0.927	0.830	0.929	0.864	0.846	0.894	0.929	0.933	0.926	0.838	0.900	1.000

**Table 3:** Compounds identified in the fruit of *C. fistula* by UHPLC/LTQ-Orbitrap MS^n^.

Peak	Lever	Compound	Mocular Formula	RT (min)	Precusor Ion *m*/*z* [M − H] ^−^	Theoretical	Error (ppm)	MS/MS Fragmentation
1b		Disaccharide	C_12_H_22_O_11_	2.963	341.10696	341.10784	2.580	**178.87711**
2b		Disaccharide	C_12_H_22_O_11_	4.273	341.10706	341.10784	2.287	**179.02390**
3	A	Unresolved	C_16_H_28_O_12_	8.613	411.14917	411.14970	1.289	341.14203, 323.08002
4	A	Unresolved	C_17_H_30_O_12_	9.907	425.16495	425.16535	0.941	407.03677, 341.14331, 323.03574
5b		Procyanidin B2	C_30_H_26_O_12_	12.320	577.13336	577.13405	1.196	559.09515, 451.17084, **425.13684, 407.19708, 289.16211, 245.02040**
6a		(+)-Catechin	C_15_H_14_O_6_	13.057	289.07065	289.07066	0.035	**244.97168, 205.01669**, 178.88663, **136.94279, 124.91268**
7	B	Unresolved	C_27_H_30_O_15_	14.765	593.14880	593.15010	2.192	473.15625, 431.16376
8a		(−)-Epicatechin	C_15_H_14_O_6_	16.667	289.07059	289.07066	0.242	**244.99500, 205.00952**, 178.94473, **136.98108, 124.88078**
11a		Rutin	C_27_H_30_O_16_	29.601	609.14343	609.14501	2.594	**301.07007**
12b		New compound	C_48_H_48_O_25_	31.840	1023.23633	1023.24009	3.675	**861.31323, 699.19556, 537.11163**
13b		Isoquercitrin	C_21_H_20_O_12_	32.917	463.08633	463.08710	1.663	**301.02444**
14b		1-[1,5-dihydroxy-3-methyl-8-[3,4,5-trihydroxy-6-[(3,4,5-trihydroxyoxan-2-yl)oxymethyl]oxan-2-yl]oxynaphthalen-2-yl]ethanone	C_24_H_30_O_13_	34.607	525.15948	525.16027	1.504	**231.00241, 187.06839**
15a		Sennoside B	C_42_H_38_O_20_	36.768	861.18451	861.18727	3.205	**699.18365, 537.19580**
16	B	Unresolved	C_30_H_26_O_9_	40.097	529.14813	529.14931	2.230	511.18488, 419.10254, 393.30389, 273.17346, 255.05096, 229.12079
17a		Quercitrin	C_21_H_20_O_11_	42.038	447.0916	447.09219	1.320	**301.04932**
18a		Sennoside A	C_42_H_38_O_20_	42.479	861.18585	861.18727	1.649	**699.19208, 537.24115**
20	C	Unresolved	C_26_H_28_O_14_	52.060	563.13892	563.13953	1.083	298.88858, 254.97116
23	C	Unresolved	C_22_H_38_O_13_	67.345	509.22232	509.22287	1.080	425.15247, 407.11340, 305.01782
24b		An isomer of kaempferol	C_15_H_10_O_6_	68.709	285.03912	285.03936	0.842	267.08939, 241.06897, 217.02310, 199.07523, 174.97690, 150.96159
25b		1,3,8-Trihydroxy-6-methoxyanthraquinone	C_15_H_10_O_6_	73.540	285.03943	285.03936	0.246	**270.00009, 240.98254**, 226.09625
26b		9-[2-carboxy-4-hydroxy-10-oxo-5-[(2S,3R,4S,5S,6R)-3,4,5-trihydroxy-6-(hydroxymethyl)oxan-2-yl]oxy-9H-anthracen-9-yl]-4,5-dihydroxy-10-oxo-9H-anthracene-2-carboxylic acid	C_36_H_28_O_15_	74.393	699.13239	699.13445	2.946	**655.15094, 537.12469**
27a		Naringenin	C_15_H_12_O_5_	76.180	271.06003	271.06010	0.258	**176.96495, 150.87328, 118.95908, 92.97495**
28a		Kaempferol	C_15_H_10_O_6_	79.519	285.03922	285.03936	0.491	**257.07129**, 240.93329, **210.97031**
29	D	Unresolved	C_30_H_45_O_10_	85.477	564.29437	564.29290	2.605	520.34570, 301.09589, 289.14099, 227.13184
30a		Rhein	C_15_H_8_O_6_	89.860	283.02362	283.02371	0.318	**256.99457, 238.92233**
31b		1-O-Methylchrysophanol	C_15_H_10_O_5_	96.553	269.04443	269.04445	0.074	**253.97797, 225.87038**
32	D	Unresolved	C_18_H_32_O_4_	98.540	311.22028	311.22169	4.531	261.02310, 201.12375
33a		Emodin	C_15_H_10_O_5_	102.796	269.04431	269.04445	0.520	**240.99141, 224.98187**
34	D	Unresolved	C_17_H_26_O_3_	105.780	277.17957	277.17982	0.902	233.14291, 205.13834
35	D	Unresolved	C_19_H_34_O_6_	106.893	357.22681	357.22717	1.008	329.25873
36	D	Unresolved	C_15_H_22_O_2_	112.957	233.15359	233.15361	0.086	218.13152, 164.77550, 146.82094

The ions in bold values were diagnostic ions. In the “Peak” of the above table, a: Components were confirmed by comparison with reference standards; b: Structures were tentatively inferred. In the “Lever” of the above table, it was interesting to classify unknown compounds into four levels by spectral similarity, they were respectively A, B, C and D.

**Table 4:** Factor loading matrix of tested samples.

Peak	Principal Component Values	
	PC1	PC2
a	−0.099	−0.493
b	−0.300	0.705
c	0.555	0.735
d	−0.351	0.334
f	0.214	0.395
g	0.987	0.056
i	0.295	0.383
j	0.937	0.218
l	0.916	0.278
m	0.782	−0.517
r	0.263	0.514
s	0.233	0.420
t	0.726	0.609
u	0.423	0.611
v	−0.156	−0.281

## Supplementary Materials

The following are available online. Table S1: The peak area of the fifteen common peaks; Figure S1: The chromatogram of secondary fragment ions about Compound **12**; Figure S2: The chromatogram of secondary fragment ions about Compound **26**; Figure S3: The chromatogram of secondary fragment ions about Compound **14**; Figure S4: The chromatogram of tertiary fragment ions about Compound **14**.
