# Supplementary material for: Chemical Profiling and Screening of the Marker Components in the Fruit of Cassia fistula by HPLC and UHPLC/LTQ-Orbitrap MSn with Chemometrics
Source: Molecules. 2018 Jun 21;23(7):1501. doi: 10.3390/molecules23071501 (PMC6100387; doi:10.3390/molecules23071501)
Supplement: Supplementary file 1 [file molecules-23-01501-s001.pdf]

Chemical Profiling and Screening of the Marker Components in the Fruit of *Cassia fistula* by HPLC and UHPLC/LTQ-Orbitrap MS^n with Chemometrics

Supplementary Materials

Table 1. The peak area of the fifteen common peaks.

Peak	RT(min)	S1	S2	S3	S4	S5	S6	S7	S8	S9	S10	S11	S12	R
1	4.35	149828	61927	115555	68219	75086	70961	73517	57580	64750	36023	46053	58539	73170
2	10.39	61513	178430	77870	211018	414681	131677	218175	226552	234782	227217	336120	347247	222107
3	14.34	26250	44441	83924	83337	132366	25886	111181	148898	73208	143826	31955	60176	80454
4	17.34	67390	44398	78938	65913	91725	36779	52787	59534	61020	32390	67185	54586	59387
5	28.43	63350	42650	18395	44666	189914	96711	81641	115523	117787	112588	47657	70730	83468
6	34.54	118262	37366	206434	54658	65838	172294	87563	441440	69636	303503	32051	55738	137065
7	39.99	46001	35773	63349	32611	63566	47117	64472	83602	60871	34384	55069	49742	53046
8	44.76	110470	53140	325709	75495	93637	147536	88450	463469	87996	504153	80878	98171	177426
9	52.01	19018	6030	119918	20207	15519	36438	41222	166328	17484	182485	21461	27566	56140
10	53.38	304826	147249	6811	25158	19175	385397	51880	446572	34912	293075	21938	42356	148279
11	68.63	64681	58285	49559	79391	206286	80046	85340	161137	103674	100299	63856	81708	94522
12	76.19	54206	72906	20461	84347	86525	76240	188376	138778	86051	118331	91265	94308	92649
13	88.87	111588	111461	251535	165941	229190	147709	298503	386549	232463	333435	121936	182831	214429
14	94.20	18994	27285	90762	62671	74895	32124	217538	146779	72137	132509	44007	67592	82275
15	111.58	15428	23115	14714	16697	15355	12713	13526	14868	11839	13505	15880	9896	14795

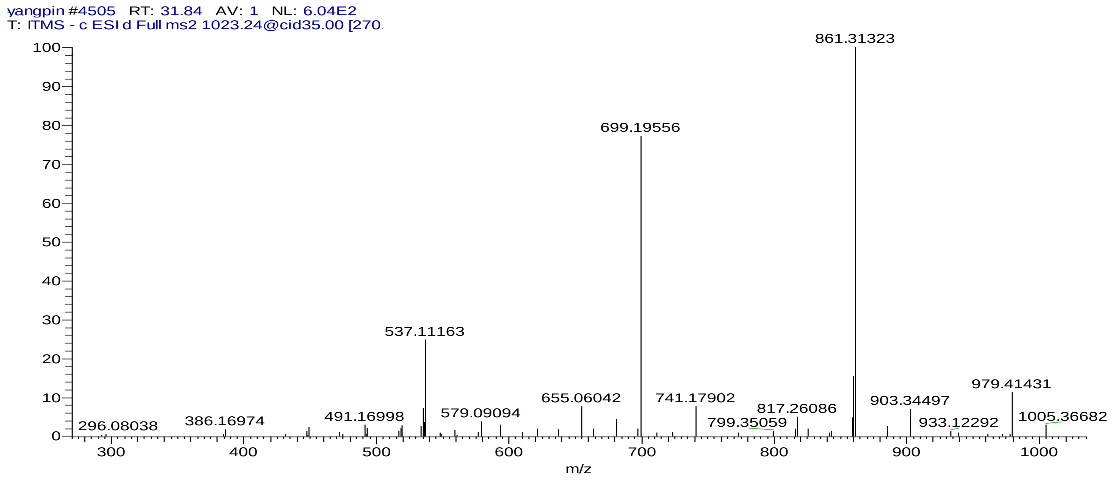

Figure 1. The chromatogram of secondary fragment ions about Compound **12**.

(sennoside aglycon linked with three molecules of glucose).

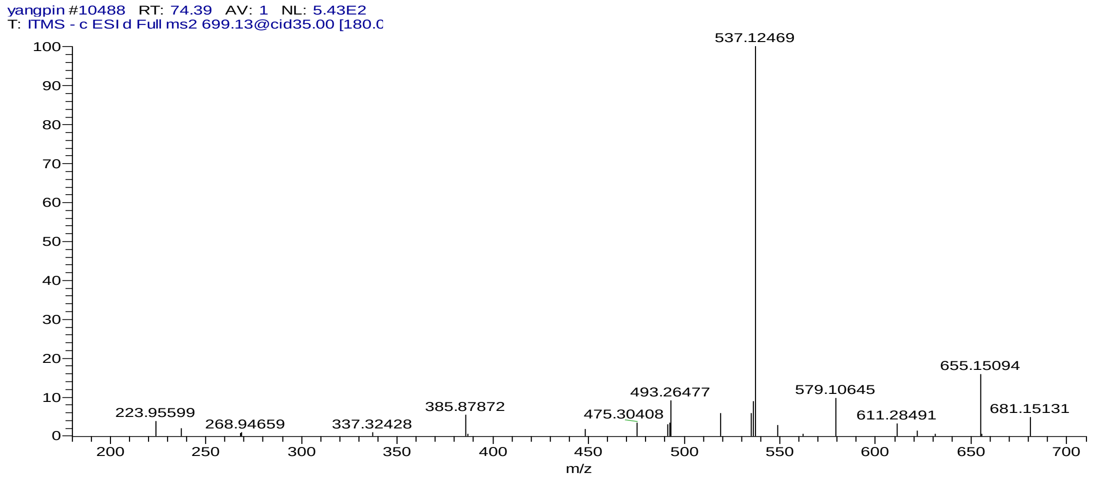

Figure 2. The chromatogram of secondary fragment ions about Compound **26**.

(sennoside aglycon linked with one molecule of glucose, 9-[2-carboxy-4-hydroxy-10-oxo-5-[(2S,3R,4S,5S,6R)-3,4,5-trihydroxy-6-(hydroxymethyl)oxan-2-yl]oxy-9H-anthracen-9-yl]-4,5-dihydroxy-10-oxo-9H-anthracene-2-carboxylic acid).

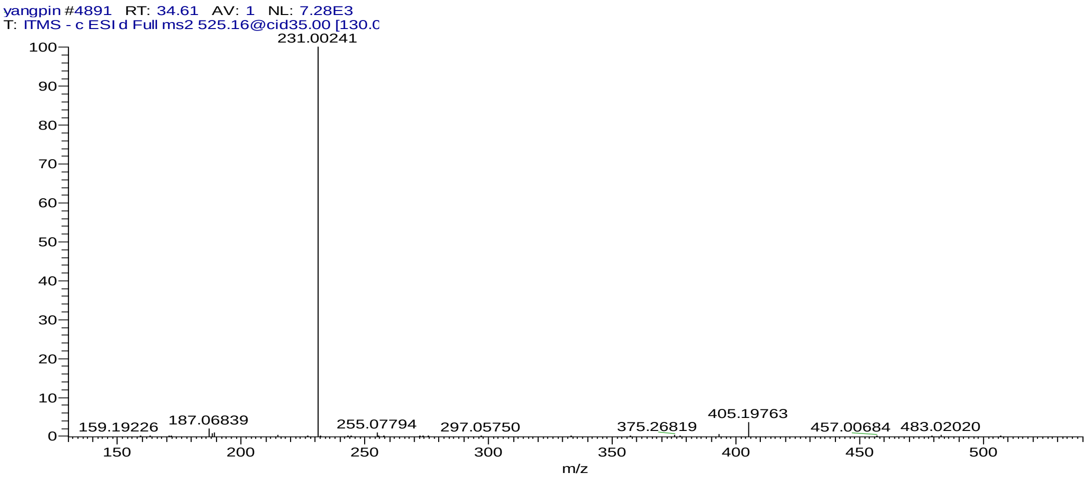

Figure 3. The chromatogram of secondary fragment ions about Compound **14**.

(1-[1,5-dihydroxy-3-methyl-8-[3,4,5-trihydroxy-6-[(3,4,5-trihydroxyoxan-2-yl)oxymethyl]oxan-2-yl]oxynaphthalen-2-yl]ethanone).

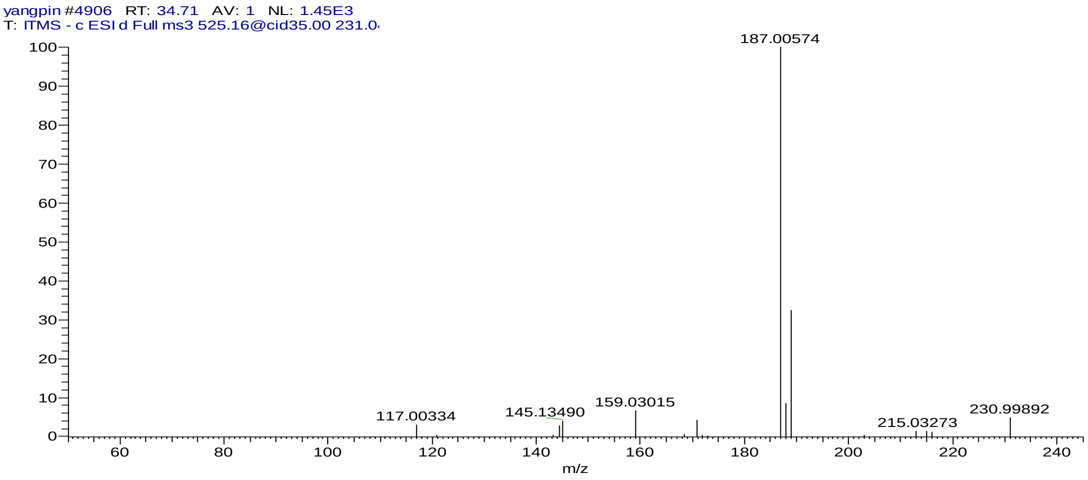

Figure 4. The chromatogram of tertiary fragment ions about Compound **14**.

(1-[1,5-dihydroxy-3-methyl-8-[3,4,5-trihydroxy-6-[(3,4,5-trihydroxyoxan-2-yl)oxymethyl]oxan-2-yl]oxynaphthalen-2-yl]ethanone).
